# Using Computer Modeling and Experimental Methods to Screen for Aptamers That Bind to the VV-GMCSF-LACT Virus

**DOI:** 10.3390/molecules29225424

**Published:** 2024-11-17

**Authors:** Maya Dymova, Natalia Vasileva, Daria Malysheva, Alisa Ageenko, Irina Shchugoreva, Polina Artyushenko, Felix Tomilin, Anna S. Kichkailo, Elena Kuligina, Vladimir Richter

**Affiliations:** 1 The Laboratory of Biotechnology, Institute of Chemical Biology and Fundamental Medicine SB RAS, 630090 Novosibirsk, Russia; nataly_vas@bk.ru (N.V.); d.malysheva@g.nsu.ru (D.M.); a.ageenko@alumni.nsu.ru (A.A.); kuligina@niboch.nsc.ru (E.K.); richter@niboch.nsc.ru (V.R.); 2 The Laboratory For Biomolecular and Medical Technologies, Krasnoyarsk State Medical University Named after Professor V.F. Voyno-Yasenetsky, 660022 Krasnoyarsk, Russia; shchugorevai@mail.ru (I.S.); art_polly@mail.ru (P.A.); annazamay@yandex.ru (A.S.K.); 3 The Laboratory for Digital Controlled Drugs and Theranostics, Federal Research Center “Krasnoyarsk Science Center of the Siberian Branch of the Russian Academy of Sciences”, 660036 Krasnoyarsk, Russia; felixnt@gmail.com; 4 The Laboratory for Magnetic Phenomena, L.V. Kirensky Institute of Physics SB RAS, 660036 Krasnoyarsk, Russia

**Keywords:** aptamer, oncolytic virus VV-GMCSF-Lact, microscale thermophoresis, cytofluorimetry, molecular dynamics, quantum chemical calculations, FMO-DFTB

## Abstract

Oncolytic virotherapy is a promising approach for cancer treatment. However, when introduced into the body, the virus provokes the production of virus-neutralizing antibodies, which can reduce its antitumor effect. To shield viruses from the immune system, aptamers that can cover the membrane of the viral particle are used. Aptamers that specifically bind to the JX-594 strain of the vaccinia virus were developed earlier. However, the parameters for binding to the recombinant virus VV-GMCSF-Lact, developed based on the LIVP strain of the vaccinia virus, may differ due its different repertoire of antigenic determinants on its membrane compared to JX-594. In this work, the spatial atomic structures of aptamers to JX-594 and bifunctional aptamers were determined using molecular modeling. The efficiency of viral particles binding to the aptamers (EC50), as well as the cytotoxicity and stability of the aptamers were studied. The synergistic effect of the VV-GMCSF-Lact combination with the aptamers in the presence of serum was investigated using human glioblastoma cells. This proposed approach allowed us to conduct a preliminary screening of sequences using in silico modeling and experimental methods, and identified potential candidates that are capable of shielding VV-GMCSF-Lact from virus-neutralizing antibodies.

## 1. Introduction

Aptamers are short, single-stranded DNA or RNA oligonucleotides that range in size from 20 to 60 nucleotide bases, and are characterized by a low molecular weight, specificity, low immunogenicity, high affinity for the target, and low toxicity [1]. The primary sequence is typically found using various SELEX procedures, where a variety of molecules, viruses, and cells are used as ligands [2]. The recombinant virus VV-GMCSF-Lact is undergoing clinical trials as an antitumor drug for breast cancer (www.clinicaltrials.gov/, NCT05376527, accessed on 15 October 2024). It is worth noting that these clinical trials use intratumoral administration of the oncolytic virus. Its cytotoxicity against various tumor cell lines and antitumor effects have been previously demonstrated, including on human glioblastoma xenograft models [3]. Despite the possibility of intrathecal administration to create the maximum concentrations of the drug in brain tissue and cerebrospinal fluid, it would be interesting to study the possibility of using intravenous administration. A serious obstacle to such administration may be the circulating neutralizing antibodies in the bodies of people that were previously vaccinated against smallpox, i.e., in people born before 1980 [4]. According to a study on people vaccinated before 1980 and born before 1975, approximately 59.2% have antibodies with neutralizing activity against VACV [4]. It is important to note that some countries continue to use the VACV vaccine. Interestingly, the level of antibodies in individuals vaccinated only once at birth is similar to those who received the vaccine twice—once at birth and again at age 8 [5].

Viruses can be shielded from the immune system using aptamers, which can coat the shell of the viral particle. Previously, using the example of vesicular stomatitis virus, effective shielding by aptamers against neutralizing antibodies was demonstrated [6]. The VV-GMCSF-Lact virus was developed based on the LIVP VACV strain of the vaccinia virus, so one of the objectives of this work was to select the most promising aptamer candidates from the pool that had been previously obtained using the SELEX procedure for the JX-594 strain of the vaccinia virus [7]. The initial aptamers were 80 nucleotides long and included primer regions that are 20 nucleotides long at the 5′ and 3′ ends, which were necessary for PCR amplification during selection. As a rule, the central part of the aptamer is involved in binding to the target, since it is selected for during the SELEX procedure, and the primer regions can also partially participate in the formation of the spatial structure necessary for the formation of the aptamer binding site with the target. Thus, optimization of the aptamer structure by removing non-functional regions while maintaining the features of its original spatial structure can lead to a decrease in non-specific binding and an increase in its affinity [8]. It is also worth noting that shortening the aptamer reduces its cost of synthesis. We have previously shown that one of the aptamers to JX-594 (NV14t_56) effectively bound to the VV-GMCSF-Lact virus, and no aggregation of viral particles was observed [9]. In this work, the screening of truncated aptamers to JX-594 was continued using biophysical and biochemical methods [7]. Additionally, to increase the efficiency of targeted delivery of the oncolytic virus to glioblastoma cells, we designed and characterized new bifunctional aptamers that, on the one hand, bind to VV-GMCSF-Lact and, on the other hand, are tropic to gliomas. Therefore, the aim of this work was to select the most promising candidates for the creation of drugs based on aptamers to increase the antitumor efficacy of the oncolytic virus VV-GMCSF-Lact using computer modeling and experimental methods.

## 2. Results

### 2.1. Modeling of the Aptamer Structure

**Figure 1:**
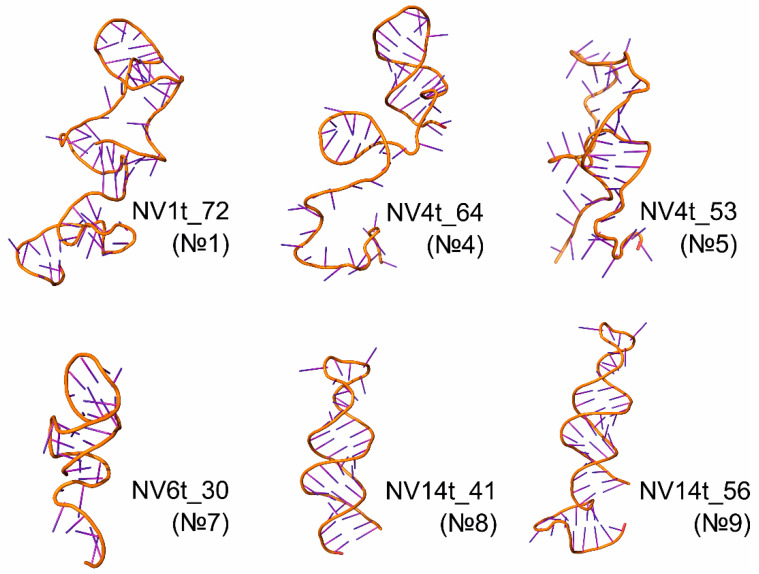
Spatial structures of aptamers that specifically bind to the JX-594 strain of the vaccinia virus.

Modeling of the aptamer structure was performed on the aptamers that exhibit specific binding affinity for the JX-594 strain of the vaccinia virus and had previously been shown to effectively bind to VV-GMCSF-Lact [10]. The tertiary structure of these aptamers was modeled based on their previously developed shortened sequences. In this work, molecular dynamics calculations were performed on all these aptamer models, followed by a cluster analysis of the trajectories and optimization of the geometry of the obtained structures using quantum chemical methods. The resulting spatial structures are presented in Figure 1.

The analysis of molecular dynamic trajectories allows us to estimate the conformational flexibility of aptamers in solution under conditions that simulate the environment of in vitro experiments. Among the considered aptamers, aptamer NV4t_64 (№4), which has a long stretch of unpaired nucleotides at the 5′ end, had the greatest lability. The “branched” form of aptamer NV1t_72 (№1) is due to the formation of three loops, whose structure was stabilized by segments of complementary nucleotide pairing. In contrast, the aptamers contain one or two loops in their structure, so they take a compact “linear” form in solution.

**Figure 2:**
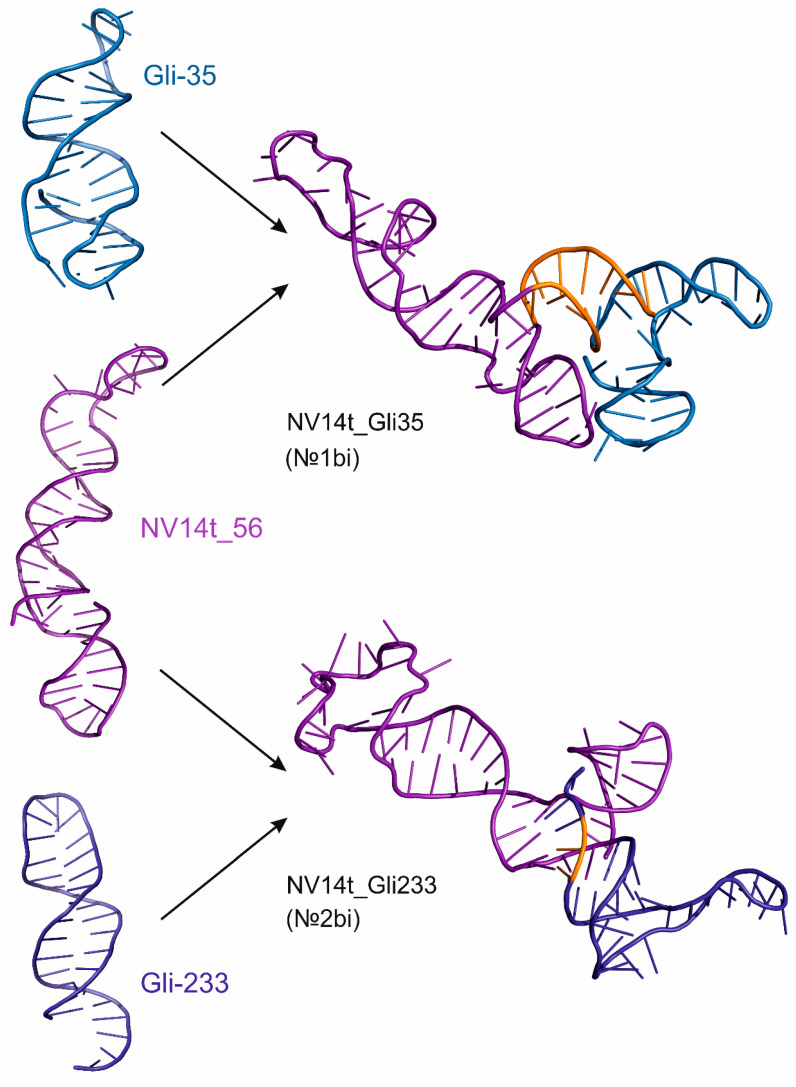
Spatial structures of initial aptamers Gli-233, Gli-35 (blue color), and NV14t_56 (purple color) and bifunctional aptamers based on them. Nucleotides that were not part of the original aptamers and were added for the stability of the atomic structure of the hybrids are highlighted in orange.

Among the aptamers to JX-594, NV14t_56 (№9) bound most effectively to VV-GMCSF-Lact, without causing the aggregation of viral particles [9]. Therefore, for the modeling of bifunctional hybrid aptamers, the truncated aptamer №9 and the glial tumor-specific aptamers Gli-233 and Gli-35 [11] were selected. The spatial structures of the original oligonucleotides are shown in Figure 2 on the left, and the hybrid aptamers are shown on the right. The color scheme of the bifunctional aptamers allows the conservation of the original aptamer structure to be evaluated in hybrid aptamers. Nucleotides added to maintain the given spatial structure of the bifunctional aptamer, which were not included in the sequence of the original №9, Gli-233, and Gli-35 sequences, are shown in orange.

To connect aptamers №9 and Gli-35 to their original sequences, an additional twelve nucleotides were added to form a “connecting bridge” between them. The initial selection of nucleotides to preserve the structural motifs of the initial aptamers №9 and Gli-35 was carried out in the mFold program. As a result, aptamer NV14t_Gli35 (№1bi), which contains one hundred and seven nucleotides, was modeled. From the perspective of achieving the dual functionality of an aptamer, hybrid №1bi has a suitable structure. The complementary linker not only maintains the desired spatial conformation of the individual components of the hybrid but also spatially separates these parts, preventing them from intertwining. This arrangement ensures that all components of the aptamer remain accessible for interaction with the target (Figure 2).

The hybrid aptamer NV14t_Gli233 (№2bi) was generated by combining the sequences of the aptamers NV14t_56 and Gli-233, along with the incorporation of five additional nucleotides to form complementary pairs and preserve the desired conformation. In comparison to hybrid №1bi, the composition of №2bi is more closely aligned with that of the original aptamers. As illustrated in Figure 2, the hybrid aptamer №2bi also exhibits an “open” configuration, allowing all the components of the aptamer to remain accessible for interaction with the target.

### 2.2. Aptamer Stability Assessment in the Presence of Fetal Bovine Serum

The serum contains components that can potentially influence the stability of aptamers, primarily nucleases. Accordingly, an important preliminary stage in the development of drugs based on aptamers is the evaluation of their stability in the presence of serum. The stability assessment of the aptamers in the presence of 10% fetal bovine serum (FBS) was carried out according to a method that was previously described in the literature [12], with sampling after 1, 2, 5, 10, 30, and 60 min (Table 1). The analysis of the samples was carried out using denaturing urea polyacrylamide gel electrophoresis (SDS-PAGE). For the subsequent calculations of the results, two-way ANOVA was used.

Dunnett’s post hoc test showed a statistically significant difference (*p*-value ≤ 0.050) between the corresponding control points and aptamer №4 (at 10, 30, and 60 min) and aptamer №8 (at 30 and 60 min). At the same time, the stability of aptamer №8 significantly dropped after 30 min of incubation with FBS to 41 ± 3%, and to 15 ± 3% after 60 min. Since aptamer №8 was the most sensitive to the DNase activity of serum nucleases, it was not used in further experiments. When incubating bifunctional aptamers and their controls with 10% FBS, a decrease in stability to 70 ± 0% and to 61 ± 1% was observed for the scrambled controls 5bi and 6bi, respectively. Bifunctional aptamers 1bi and 2bi showed high stability against the DNase activity of serum nucleases: after 60 min of incubation, 1bi retained stability at the level of 97 ± 0%, and 2bi remained at the level of 90 ± 0%. Thus, aptamer №8 was excluded from further experiments, since it showed the greatest sensitivity to the DNase activity of serum nucleases, and the rest of the candidate aptamers were studied further.

### 2.3. Evaluation of Cytotoxicity of Aptamers Against Human Glioma Cells and hFF8 Normal Human Fibroblasts 

**Figure 3:**
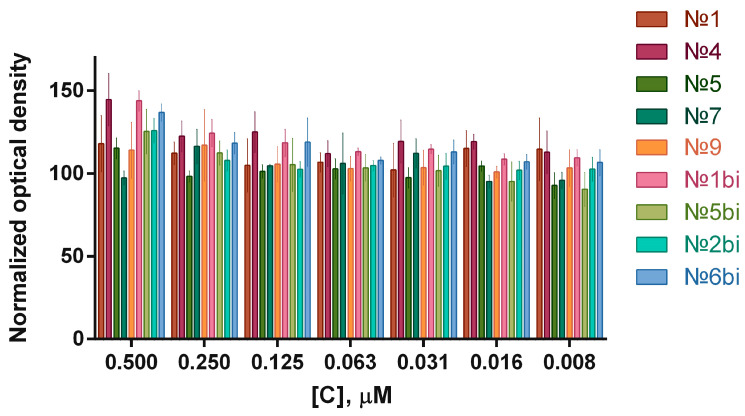
Normalized optical densities at 570 nm, which was measured 24 h after the treatment of glioma cells with aptamers in the concentration range of 0.500 μM to 0.008 μM. Error bars represent ±standard deviation.

The cytotoxicity of the aptamers that are specific to the vaccinia virus, as well as the bifunctional hybrids, against adherent human glioma cells was determined using a colorimetric test (Figure 3).

**Figure 4:**
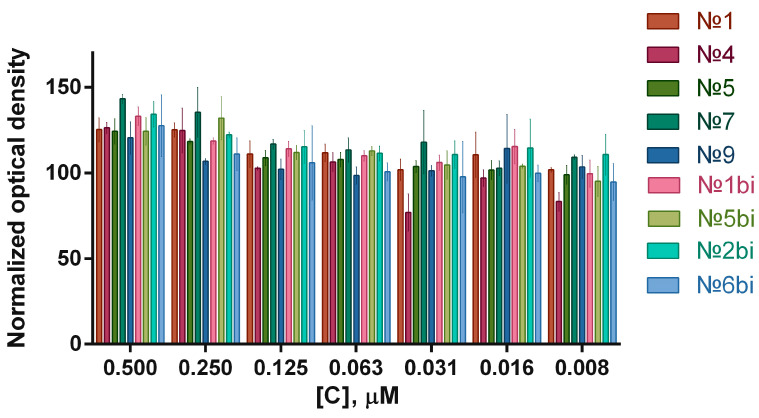
Normalized optical densities at 570 nm, which was performed 24 h after the treatment of normal human fibroblasts in with aptamers in the concentration range of 0.500 μM to 0.008 μM. Error bars represent ±standard deviation.

The aptamer concentration range was from 0.500 μM to 0.008 μM, and was chosen based on literature data: an increase in aptamer concentration above 0.500 μM may lead to an incorrect interpretation of results due to non-specific binding to cells [13]. At an aptamers concentration of 0.500 μM, there was a slight increase in the viability of tumor cells, which requires consideration in experiments to assess the cytotoxicity of VV-GMCSF-Lact in in vitro experiments. Similarly, an experiment was conducted to assess the aptamers’ cytotoxicity against hFF8 human normal fibroblast cells (Figure 4).

When incubated with aptamer № 4, even at a concentration of 0.008 M, a decrease in the viability of the normal human fibroblast cells was observed (*p* ≤ 0.0001), and therefore its use in further in vitro experiments was undesirable. In addition, the selective cytotoxicity of some aptamers (№1, 4, 5, 9, and 2bi) against neurospheres of glioma cells was analyzed to determine the general trend of the influence of the aptamers on the viability of 3D cell cultures. In the case of neurospheres, a weak cytotoxic effect of 0.500 μM of aptamer №5 was observed compared to 0.250 μM and 0.063 μM (*p*-value ≤ 0.010).

### 2.4. The Binding Efficiency of Aptamers Against the Recombinant Virus VV-GMCSF-Lact

**Figure 5:**
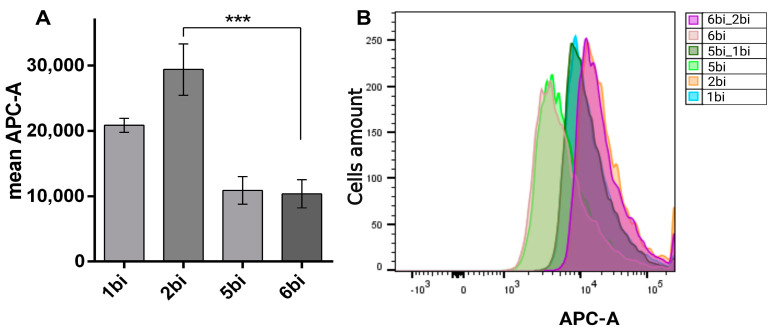
Evaluation of binding of Cy5-modified bifunctional hybrids with oncolytic virus VV-GMCSF-Lact (**A**). Distribution of cells by fluorescence intensity in the APC-A channel (**B**). *** indicates *p*-value ≤ 0.001.

The binding of Cy5-modified aptamers to the virus was evaluated using cytofluorometry (Figure 5). For aptamers to the recombinant JX-594 strain of the vaccinia virus, the binding efficiency was previously shown, and the most specific aptamers were №1, 4, 5, 8, and 9 [11]. Among the bifunctional hybrids, aptamer №2bi showed the highest binding efficiency for VV-GMCSF-Lact compared to aptamer №1bi (*p*-value ≤ 0.050). It is worth noting that the corresponding scramble control (№6bi) selected by the program “GenScript” had a statistically lower specificity of binding to the virus (*p*-value ≤ 0.001).

We estimated the effect of 30 min of blocking with the corresponding scramble controls (5bi or 6bi) on the binding of the target aptamers (1bi or 2bi) to the target VV-GMCSF-Lact. The mean fluorescence value (MFU) in the APC-A channel did not drop significantly, and remained at the same level as that of the target aptamers (MFU = 20,000–30,000). This means that the target aptamers bound to specific target epitopes, while scramble controls bound non-specifically. Since the MFU for aptamer №2bi was higher than that of aptamer №1bi (*p*-value ≤ 0.050) (i.e., №2bi showed greater binding efficiency to VV-GMCSF-Lact compared to aptamer №1bi, the bifunctional hybrid), №2bi and its corresponding scrambled control №6bi were chosen for subsequent work.

### 2.5. Using Microscale Thermophoresis to Assess Aptamer–Virus Interactions

**Figure 6:**
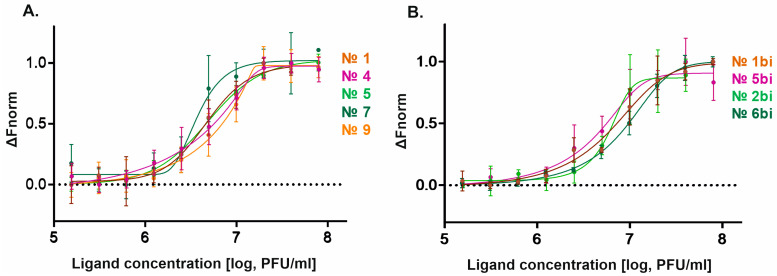
Dose–response relationships for the binding interaction between aptamers and VV-GMCSF-Lact. (**A**) Aptamers to VV-GMCSF-Lact; (**B**) bifunctional aptamers.

Next, microscale thermophoresis was used to assess the molecular interactions between the aptamers and the oncolytic virus VV-GMCSF-Lact in the concentration range of 1.56 × 10^5^ to 8.00 × 10^7^ PFU/mL. The binding curves for the aptamers are shown in Figure 6.

The half-maximal effective concentration value (EC50) was determined using the generalized Hill equation for data fitting and the following parameters: curve slope, lower and upper asymptotes, curve shift along the concentration axis, and curve asymmetry relative to the inflection point. The obtained EC50 of VV-GMCSF-Lact for the aptamers are presented in Table 2.

Two-way ANOVA was used to compare the obtained EC50 values, and Tukey’s test was used for pairwise comparisons of values. A statistically significant difference was found between aptamers №7 and №9 (*p* = 0.0207). The bifunctional aptamers were compared with the corresponding scramble controls and a statistically significant difference was shown between the bifunctional aptamer №2bi and its scramble control №6bi (*p* = 0.0033). No statistically significant difference was observed between bifunctional aptamers №1bi and №2bi.

The EC50 is the value at which half of the aptamers are in the bound state. Since a statistically significant difference was shown between the maximum and minimum values for the № 7 and №9 aptamers to VV-GMCSF-Lact, but not between the other aptamers in the sample, we did not exclude any aptamers to VV-GMCSF-Lact from the selection process based on this experiment. In the case of the bifunctional aptamers, aptamer №1bi was excluded from further studies based on these results, since it did not differ statistically from its control (№5bi). Aptamer №2bi not only differed, but also had a lower EC50 value than its control, which indicates a greater binding efficiency. Accordingly, it was this bifunctional aptamer that was used in further work.

### 2.6. Evaluation of VV-GMCSF-Lact Efficiency in the Presence of Aptamers and Serum

**Figure 7:**
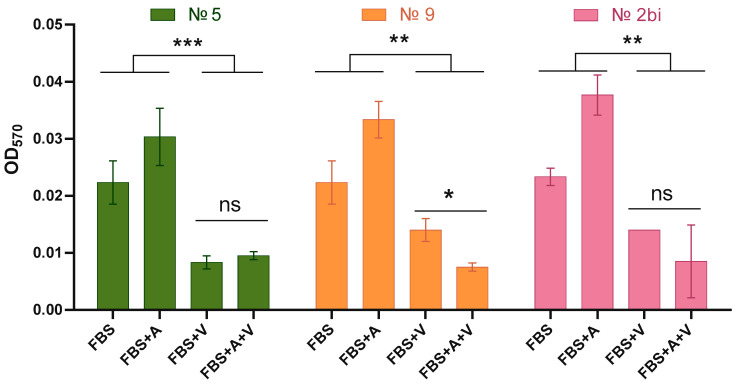
Cytotoxic effect of the oncolytic virus VV-GMCSF-Lact (V) on primary culture of human glioblastoma in the presence of aptamers (A) and serum (FBS). Three asterisks (***) indicate *p* ≤ 0.001, two asterisks (**) indicate *p* ≤ 0.01, and one asterisk (*) indicates *p* ≤ 0.05. The abbreviation “ns” denotes non-significant differences (*p* > 0.05).

To determine if there is a synergistic effect of the aptamers and an antagonistic effect of serum in combination with the oncolytic virus VV-GMCSF-Lact, primary cultures of human glioblastoma cells were treated with the following: 10% FBS; 10% FBS and VV-GMCSF-Lact at a dose of 0.1 PFU/cell; 10% FBS and 200 nM aptamers; and 10% FBS, 200 nM aptamers, and VV-GMCSF-Lact at a dose of 0.1 PFU/cell. For this evaluation, two aptamers were selected that specifically bind to VV-GMCSF-Lact, aptamers №5 and №9, as well as the bifunctional aptamer №2bi, which, according to the cytometric analysis, showed the highest binding efficiency to the oncolytic VV-GMCSF-Lact virus ([11] and the data presented in this article). It was shown that aptamer №9 had a synergistic effect (*p* ≤ 0.05) in combination with the oncolytic virus VV-GMCSF-Lact against the primary cultures of human glioblastoma cells (Figure 7). Aptamers No. 5 and 2bi did not have a synergistic cytotoxic effect with the virus; for aptamer No. 2bi, the changes showed a trend but no statistically significant differences were found between the “FBS + V” and “FBS + V + A” groups.

## 3. Discussion

Nucleic acid aptamers are short single-stranded DNA or RNA oligonucleotides that are characterized by a high binding specificity to a variety of target types, including small organic molecules, polysaccharides, proteins, viruses, bacteria, cells, and tissues [1]. They were discovered almost simultaneously by two groups of researchers about 30 years ago. Aptamers are called chemical antibodies, since they specifically bind to target molecules, similar to protein antibodies; they also possess additional advantages, including a small size, rapid and inexpensive synthesis, versatile chemical modifications allowing for improved stability and affinity, and low to no immunogenicity or toxicity [14,15,16]. Thus, aptamers can effectively replace monoclonal antibodies in a variety of applications, including immunophenotyping, immunohistochemistry, targeted drug delivery, and biosensors.

The interest of many researchers in the use of aptamers for both therapeutic and diagnostic applications has not waned [17,18]. This can be seen from both the growth of publications in the PubMed database and the number of clinical trials. Of particular interest is the use of aptamers for the development of antitumor drugs [19,20]. Increased antitumor efficacy can be achieved by immunoshielding an oncolytic virus from neutralizing antibodies, reducing the aggregation of viral particles, and using aptamers as cryoprotectants [21].

To screen the aptamers obtained from the classical SELEX procedure, it is optimal to use computer molecular modeling—both to clarify their secondary and tertiary structures and to model protein–nucleic acid interactions [22]. Tertiary structure modeling allows one to identify the nucleotides required to maintain the aptamer structure and bind to the target, and it allows one to make rational choices about shortening the original aptamer or selecting sites for tag attachment [23]. Modeling the secondary structure of aptamers that specifically bind to the JX-594 strain of the vaccinia virus was carried out earlier [10]. The corresponding tertiary structure of the aptamers was modeled and presented in this work. The molecular dynamics calculations showed that the structure of the truncated aptamers remained stable in solution under conditions corresponding to those in in vitro experiments (temperature, solvent, and ionic environment). Bifunctional aptamers were constructed by combining the previously characterized truncated aptamer №9, which binds to VV-GMCSF-Lact, with the glial tumor-specific aptamers Gli-233 and Gli-35 [11]. The hybrid aptamers were modeled in such a way that the hybrid structures retained the structural motifs of the original aptamers. The molecular dynamics calculations showed that the hybrid aptamers retained the desired structure in solution due to stable and low-mobility complementary regions.

The DNase activity of serum nucleases towards nucleic acid aptamers is the main obstacle to their use in vivo in diagnostics and therapeutic practices [12]. Therefore, an important part of aptamer development is the assessment of their responses to the action of blood serum nucleases. The screening of the obtained aptamers and hybrids for stability in the presence of fetal bovine serum revealed the aptamers most resistant to the action of serum nucleases: №1, 4, 5, 7, 9, 1bi, 5bi, 2bi, and 6bi. Since the stability of aptamer № 8 dropped after 30 min of incubation with serum to ≈40%, and to ≈14% after 60 min, it was not used in further experiments.

The evaluation of the cytotoxicity of the drugs used against transformed and normal cell lines is an integral part of any in vitro study [24]. An assessment of the cytotoxicity of the aptamers specific to the vaccinia virus, as well as the bifunctional hybrids, against adherent human glioma cells and normal human fibroblast cells was performed using a standard colorimetric test. The aptamers did not have a cytotoxic effect on the studied cell lines. It is assumed that the oncolytic virus VV-GMCSF-Lact will have a cytotoxic effect on tumor cells. It is worth noting that the aptamers at a concentration of 0.5 μM led to a slight increase in the viability of the cell cultures, which should certainly be taken into account in subsequent in vivo experiments.

The binding of aptamers to the target, the recombinant virus VV-GMCSF-Lact, was determined by cytofluorometry and microscale thermophoresis. It was previously shown that among the analyzed aptamers to JX-594, a Wyeth strain vaccinia virus, aptamers №1, 4, 5, 8, and 9 were the most specific [10], while the maximum average fluorescence intensity was observed for aptamers №1, 5, and 9. Among the bifunctional hybrids, aptamer №2bi showed the greatest binding efficiency for the recombinant virus VV-GMCSF-Lact. Since there was a statistically significant difference between it and its scrambled control (№6bi), this pair was chosen for subsequent work. Microscale thermophoresis is based on the measurement of the mobility of molecules in a temperature gradient, and allows us to quantitatively estimate the bimolecular interactions between the studied molecule and the ligand. The physical meaning of the half-maximal effective concentration (EC50) in the binding experiment using the Monolith NT.115 device is the ligand concentration at which half of the target is in the bound state [25]. According to the microscale thermophoresis results, the EC50 varied from 3.7 ± 0.6 × 10^6^ PFU/mL to 9.8 ± 0.8 × 10^6^ PFU/mL. It is worth noting that EC50 by definition always depends on the concentration of the target molecule (aptamer), i.e., it only allows you to compare ligands measured in the same experiment. With the help of microscale thermophoresis, the selection of the aptamers that most effectively bind to the virus was carried out. According to the results of the experiment, the bifunctional aptamer №1bi was excluded, since it did not show a statistically significant difference in the EC50 value compared to its control (5bi). Aptamers №1–9, 2bi, and 6bi all fulfilled the selection criterion, since a statistically significant difference was observed only between the highest and lowest values, but not between the other aptamers.

The evaluation of the synergistic effect of aptamers and the antagonistic effect of serum in combination with the oncolytic virus VV-GMCSF-Lact against primary human glioblastoma cells identified two candidate aptamers, №9 and № 2bi, which could be used in further experiments. It was shown that aptamer №9 had a synergistic effect (*p* ≤ 0.05) in combination with VV-GMCSF-Lact against primary human glioblastoma cells. For aptamer № 2bi, the changes only showed a trend; however, considering its binding efficiency to the oncolytic virus, shown in this study, and to immortalized human glioblastoma cells, as shown previously [10], we also consider it a promising candidate for the development of an antitumor drug based on the recombinant virus VV-GMCSF-Lact.

## 4. Materials and Methods

### 4.1. Cell Cultures

The primary tumor cells from a human glioblastoma were obtained by mechanical and enzymatic destruction of a glioblastoma (G4 according to WHO) that was resected during surgery in a patient in 2022. The material was collected with the approval of the Local Ethics Committee (LEC) of the Federal State Budgetary Educational Institution of Higher Education “Krasnoyarsk State Medical University named after Professor V.F. Voyno-Yasenetsky” of the Ministry of Health of the Russian Federation (Protocol No. 95/2020, dated 29 January 2020), as well as with the approval of the bioethics commission of the LEC of KrasSMU (dated 5 November 2019) and the LEC of the “Krasnoyarsk Interdistrict Clinical Emergency Hospital named after N.S. Karpovich” (dated 20 November 2016). hFF8 normal human fibroblasts were kindly provided by Dr. Filipenko M.L. (Laboratory of Pharmacogenomics of the Institute of Chemical Biology and Fundamental Medicine SB RAS). Cultures of the glioblastoma cells and hFF8 normal fibroblasts were cultured in DMEM/F12 and IMDM media (Gibco, Waltham, MA, USA), respectively, supplemented with 10% FBS, 2 mM L-glutamine, 1× solutions of essential amino acids MEM, and antibiotic–antimycotic (Gibco, Waltham, MA, USA), and grown at 37 °C in a CO_2_ incubator.

### 4.2. Molecular Modeling

Bifunctional hybrid aptamers (Table 3) were constructed based on the original sequence of the NV14t_56 aptamer, which is specific to VV-GMCSF-Lact [9,10], and the Gli35 and Gli233 aptamers, which specifically bind to human glioblastoma cells [11]. The aptamers were synthesized in the Laboratory of Synthetic Biology of the Institute of Chemical Biology and Fundamental Medicine SB RAS.

The secondary structures of the aptamers were simulated using mFold, which determines nucleic acid folding patterns based on nucleotide sequence data [26].The spatial atomic structures of the aptamers were constructed using the SimRNA [27] and VMD [28,29] programs. Molecular dynamics calculations were performed in the GROMACS 2018.8 software package [30] using the Amber14sb force field [31] and the TIP3P water model [32].The aptamers were solvated under periodic conditions in a cubic box. Sodium (Na^+^) counterions were introduced to neutralize the negative charges of the aptamers, while Na^+^ and chloride (Cl^−^) ions were added to create a background electrolyte, achieving a total salt concentration of 0.15 M. The cluster analysis of the obtained molecular dynamics trajectories was conducted using the VMD program [33].

For the obtained representative structures, geometry optimization was performed using the FMO2-DFTB quantum chemical method [34] with 3ob-3-1 parameters [35] within the framework of the polarizable continuum solvent model (C-PCM) with the Grimm dispersion correction D3(BJ) [36] implemented in the GAMESS program [37,38].

Sequences of randomized scrambled aptamers Scrbl_NV14t_Gli35 (№5bi) and Scrbl_Gli233_NV14t (№6bi) were selected using the GenScript program (www.genscript.com/tools/create-scrambled-sequence, accessed on 1 February 2024). This software creates a scrambled sequence as a negative control for experiments, which has an identical nucleotide composition to the input sequence (between NV14t_Gli35 (№1bi) and between NV14t_Gli233(№2bi), respectively), and undergoes the same types of filtering. The aptamers from Ref. [10] were also used in this work, namely NV1t_72 (№1), NV4t_64 (№4), NV4t_53 (№5), NV6t_30 (№7), NV14t_41 (№8), and Nv14t_56 (№9).

### 4.3. Stability of Aptamers in the Presence of Fetal Bovine Serum

The stability of the aptamers was studied using a previously described method with minor modifications [12]. The renatured aptamer was incubated in 10% FBS (Capricorn Scientific GmbH, Ebsdorfergrund, Germany) in IMDM culture medium (Sigma, St. Louis, MO, USA) for 1 h at 37 °C. At the indicated time points (immediately after addition (control), 1 min, 2 min, 5 min, 10 min, 30 min, and 1 h), 10 pmol aliquots of the mixture were taken. Stop buffer (8 M urea and 0.01% bromophenol blue) was added to the aliquots and stored at −20 °C. The analysis was performed using denaturing polyacrylamide gel electrophoresis (15%). Visualization was achieved using Cy5 fluorescence at a wavelength of 600 nm and an Amersham Typhoon FLA 9500 fluorescence scanner (Cytiva, Uppsala, Sweden). Quantitative assessment of band intensity was performed using the Quantity One program (Bio-Rad, Hercules, CA, USA). The relative stability of the aptamer was calculated using the following formula: [100% × (band intensity corresponding to the intact aptamer)/(sum of intensities of all bands in the lane)], using global background subtraction.

### 4.4. Cytotoxic Activity of Aptamers on Human Glioma Cells

To determine the cytotoxicity of the aptamers against primary cultured human glioblastoma cells, a previously described protocol with minor modifications was used [39,40,41]. A total of 6000 cells in 100 μL of Opti-MEM, containing 2 mM L-glutamine, 1× antibiotic-antimycotic solution (Gibco, Waltham, MA, USA) were added to the wells of a 96-well plate. The filled plate was incubated for 72 h at a temperature of 37.0 ± 1.0 °C in an atmosphere of 5.0 ± 0.5% CO_2_. After 72 h, eight dilutions of aptamers (0.5, 0.25, 0.125, 0.0625, 0.0313, 0.0156, and 0.0078 μM) were prepared in Opti-MEM, containing 2 mM L-glutamine, and 1× antibiotic–antimycotic solution. Then, the plate was incubated for 24 h at 37.0 °C in a humidified atmosphere of 5.0% CO_2_. After 24 h of incubation, 20 μL of the reagent from the Deep Blue Cell Viability™ Kit (BioLegend, Inc., San Diego, CA, USA) was added to each well and incubated for 4–8 h at 37 °C. The optical density of the solution was estimated using a colorimetric detection method and an Apollo-8 Microplate Absorbance Reader LB 912 spectrophotometer (Berthold Technologies, Bad Wildbad, Germany). The background absorption at 620 nm was subtracted from the absorption of resorufin at 590 nm. Cell viability was determined relative to the viability of control cells (100%) ± SD (standard deviation) based on the results of three independent experiments.

### 4.5. The Binding Efficiency of Aptamers to the Oncolytic Virus VV-GMCSF-Lact Using Cytofluorometry

Assays of the binding of Cy5-modified aptamers to the virus were performed according to a protocol described previously, with minor modifications [6]. VV-GMCSF-Lact (10^7^ PFUs) was incubated with 2.5 μL of yeast RNA (2 mg/mL) for 30 min at 25 °C on a shaker to block non-specific binding. Then, the virus suspension was incubated for 30 min at 25 °C with 50 μL of 200 nM pre-renatured, Cy5-modified aptamers. Then, the samples were resuspended in 300 μL of DPBS and analyzed using a BD FACSCanto™ II Flow Cytometer (Becton Dickinson, Franklin Lakes, NJ, USA). Each experiment was carried out with three technical replicates.

### 4.6. VV-GMCSF-Lact Efficacy in the Presence of Aptamers and Serum

To determine the effect of the combined use of the aptamers and VV-GMCSF-Lact on the proliferation of primary human glioblastoma culture, the Deep Blue Cell Viability™ Kit assay was used. A total of 6000 cells in 100 µL of Opti-MEM containing 2 mM L-glutamine and 1× antibiotic solution (100 U/mL penicillin and 100 mg/mL streptomycin sulfate) were added to the wells of a 96-well plate. After 72 h, various combinations of 0.1 PFU/cell VV-GMCSF-Lact, 200 nM aptamer, and 10 µL of FBS (final dilution 1:10) were added and incubated for another 72 h. Then, 20 µL of the Deep Blue Cell Viability™ Kit reagent was added and the plate was incubated for 4–8 h at 37 °C. The optical density of the samples was measured at a wavelength of 570 nm and a reference wavelength of 620 nm using a microplate reader (Apollo-8 Microplate Absorbance Reader LB 912 (Berthold Technologies GmbH & Co. KG, Bad Wildbad, Germany)).

### 4.7. Microscale Thermophoresis (MST)

Aptamer binding to VV-GMCSF-Lact viral particles was measured by microscale thermophoresis [42]. Each mixture contained 0.05% Tween 20 (neoFroxx GmbH, Einhausen, Germany) to minimize any non-specific interactions. Several concentrations of viral particles were obtained through serial dilutions in 1 mM Tris HCl pH 8.5: 1.56 × 10^5^ to 8.00 × 10^7^ PFU/mL. Then, the renatured aptamer was added to each sample to achieve a final concentration of 20 nM. Measurements were performed using standard capillaries and a Monolith NT.115 device (NanoTemper Technologies GmbH, Munich, Germany). All calculations were based on three independent experiments using GraphPad Prism 6.01. For the calculation of the EC50 and slope coefficient (Hill slope), a standard dose–response curve was used, and a four-parameter logistic equation was used to determine the lower and upper plateaus of the curve.

### 4.8. Statistical Analysis

Quantitative variables are presented as the mean ± standard deviation (SD). Each experiment was repeated at least three times. Statistical analysis was performed using GraphPad 6.01 (GraphPad Software, San Diego, CA, USA). Two-way ANOVA was used to compare more than two data sets. Differences were considered significant if the *p*-value was <0.050.

## 5. Conclusions

The aim of this work was to screen aptamers, which had been previously selected using the SELEX selection technology, using molecular modeling, electrophoretic analysis, a colorimetric test for assessing proliferation, cytofluorometry, and microscale thermophoresis. The most promising candidates for use in the development of an antitumor drug based on the recombinant virus VV-GMCSF-Lact were identified: aptamer № 9, as well as bifunctional aptamer № 2bi. The presented screening design helps in selecting the nucleic aptamers with the greatest stability and efficiency of binding to the target, and the lowest cytotoxicity and aggregation ability by means of simple and inexpensive experiments. Previous theoretical modeling allowed us to determine the tertiary structure and predict the sites necessary for binding.

## Figures and Tables

**Table 1:** Stability of aptamers that specifically bind to the JX-594 strain of the vaccinia virus and bifunctional hybrids in the presence of 10% FBS.

Aptamer		Time (min)					
№	Name	1	2	5	10	30	60
1	NV1t_72	100 ± 4	101 ± 3	103 ± 6	99 ± 5	88 ± 4	76 ± 3
4	NV4t_64	98 ± 0	99 ± 2	99 ± 2	93 ± 0	76 ± 1	62 ± 2
5	NV4t_53	99 ± 3	97 ± 4	96 ± 3	93 ± 3	81 ± 5	65 ± 7
7	NV6t_30	101 ± 1	100 ± 0	101 ± 2	95 ± 1	89 ± 4	70 ± 7
8	NV14t_41	100 ± 3	100 ± 2	93 ± 7	80 ± 2	41 ± 3	15 ± 4
9	Nv14t_56	100 ± 1	98 ± 0	96 ± 1	90 ± 1	76 ± 2	59 ± 5
1bi	NV14t_Gli35	100 ± 0	100 ± 0	100 ± 0	100 ± 0	99 ± 0	97 ± 0
5bi	Scrbl_NV14t_Gli35	100 ± 1	100 ± 0	98 ± 1	95 ± 1	83 ± 1	70 ± 0
2bi	NV14t_Gli233	102 ± 0	101 ± 1	101 ± 1	100 ± 1	99 ± 0	90 ± 0%
6bi	Scrbl_NV14t_Gli233	99 ± 1	99 ± 0	96 ± 0	91 ± 0	76 ± 1	61 ± 1

**Table 2:** Half-maximal effective concentrations of VV-GMCSF-Lact for aptamers.

Aptamer	EC50 (PFU/mL)	Adjusted R^2^
№1	4.7 ± 0.8 × 10^6^	0.92
№4	5.0 ± 1.2 × 10^6^	0.93
№5	4.9 ± 0.4 × 10^6^	0.98
№7	3.7 ± 0.5 × 10^6^	0.89
№9	6.7± 1.1 × 10^6^	0.92
№1bi	7.1 ± 1.2 × 10^6^	0.94
№5bi	4.9 ± 1.0 × 10^6^	0.90
№2bi	6.2 ± 1.0 × 10^6^	0.87
№6bi	9.8 ± 0.8 × 10^6^	0.98

R^2^—adjusted coefficient of determination.

**Table 3:** Nucleotide sequences of bifunctional aptamers.

Aptamer	Nucleotide Sequence
NV14t_Gli35 №1bi *	5′-GCGTTATTAACGGAGCAGTCCTGTGGAGTGGGTGATTTACGGTAACCACGCCATCACCCTATTATCTCATTATCTCGTTTTCCCTATGCGGCATAGGTCGTAAATCA-3′
NV14t_Gli233 №2bi *	5′-ACTCGATTCCACTGCAACAACTGAACGGACTGGAATCGGTAACCACGCCATCACCCTATTATCTCATTATCTCGTTTTCCCTATGCGGCATAGGT-3′
Scrbl_NV14t_Gli35 №5bi *	5′-GCGTTATTAACGGAGCAGTCCTGTGGAGTGGGTGATTTACGGTAACCACGCCATCACCCTATTATCTCATTATCTCGTTTTCCCTATGCGGCATAGGTCGTAAATCA-3′
Scrbl_NV14t_Gli233 №6bi *	5′-ACTCGATTCCACTGCAACAACTGAACGGACTGGAATCGGTAACCACGCCATCACCCTATTATCTCATTATCTCGTTTTCCCTATGCGGCATAGGT-3′

* The aptamer was designated as this name in the article.

## Data Availability

Data are contained within the article.

## References

[1] Yan B., Li Y., He S. (2024). Aptamer-mediated therapeutic strategies provide a potential approach for cancer. Int. Immunopharmacol..

[2] Mahmoudian F., Ahmari A., Shabani S., Sadeghi B., Fahimirad S., Fattahi F. (2024). Aptamers as an approach to targeted cancer therapy. Cancer Cell Int..

[3] Vasileva N., Ageenko A., Byvakina A., Sen’kova A., Kochneva G., Mishinov S., Richter V., Kuligina E. (2024). The Recombinant Oncolytic Virus VV-GMCSF-Lact and Chemotherapy Drugs against Human Glioma. Int. J. Mol. Sci..

[4] Marchi S., Piccini G., Cantaloni P., Guerrini N., Zannella R., Coluccio R., Benincasa L., Solfanelli N., Remarque E.J., Viviani S. (2024). Evaluation of monkeypox- and vaccinia-virus neutralizing antibodies before and after smallpox vaccination: A sero-epidemiological study. J. Med. Virol..

[5] Riccardo V., Pablo G.-C. (2023). Neutralization Determinants on Poxviruses. Viruses.

[6] Muharemagic D., Zamay A., Ghobadloo S.M., Evgin L., Savitskaya A., Bell J.C., Berezovski M.V. (2014). Aptamer-facilitated Protection of Oncolytic Virus from Neutralizing Antibodies. Mol. Ther. Nucleic Acids.

[7] Labib M., Zamay A.S., Muharemagic D., Chechik A.V., Bell J.C., Berezovski M.V. (2012). Aptamer-Based Viability Impedimetric Sensor for Viruses. Anal. Chem..

[8] Dhiman A., Anand A., Malhotra A., Khan E., Santra V., Kumar A., Sharma T.K. (2018). Rational truncation of aptamer for cross-species application to detect krait envenomation. Sci. Rep..

[9] Dymova M.A., Malysheva D.O., Popova V.K., Dmitrienko E.V., Endutkin A.V., Drokov D.V., Mukhanov V.S., Byvakina A.A., Kochneva G.V., Artyushenko P.V. (2024). Characterizing Aptamer Interaction with the Oncolytic Virus VV-GMCSF-Lact. Molecules.

[10] Dymova M.A., Kuligina E.V., Richter V.A., Artyushenko P.V., Rogova A.V., Shchugoreva I.A., Tomilin F.N., Kichkailo A.S., Zamay T.N. (2023). Obtaining highly selective aptamers to the VV-GMCSF-Lact oncolytic virus. Theoretical and experimental approaches. Sib. Med. Rev..

[11] Kichkailo A.S., Narodov A.A., Komarova M.A., Zamay T.N., Zamay G.S., Kolovskaya O.S., Erakhtin E.E., Glazyrin Y.E., Veprintsev D.V., Moryachkov R.V. (2023). Development of DNA aptamers for visualization of glial brain tumors and detection of circulating tumor cells. Mol. Ther. Nucleic Acids.

[12] Sakovina L., Vokhtantsev I., Vorobyeva M., Vorobyev P., Novopashina D. (2022). Improving Stability and Specificity of CRISPR/Cas9 System by Selective Modification of Guide RNAs with 2′-fluoro and Locked Nucleic Acid Nucleotides. Int. J. Mol. Sci..

[13] Henri J., Bayat N., Macdonald J., Shigdar S. (2019). A guide to using nucleic acid aptamers in cell based assays. Aptamers.

[14] Tuerk C., Gold L. (1990). Systematic Evolution of Ligands by Exponential Enrichment: RNA Ligands to Bacteriophage T4 DNA Polymerase. Science.

[15] Ellington A.D., Szostak J.W. (1990). In vitro selection of RNA molecules that bind specific ligands. Nature.

[16] Kohlberger M., Gadermaier G. (2022). SELEX: Critical factors and optimization strategies for successful aptamer selection. Biotechnol. Appl. Biochem..

[17] Zhu C., Feng Z., Qin H., Chen L., Yan M., Li L., Qu F. (2024). Recent progress of SELEX methods for screening nucleic acid aptamers. Talanta.

[18] Askari A., Kota S., Ferrell H., Swamy S., Goodman K.S., Okoro C.C., Spruell Crenshaw I.C., Hernandez D.K., Oliphant T.E., Badrayani A.A. (2024). UTexas Aptamer Database: The collection and long-term preservation of aptamer sequence information. Nucleic Acids Res..

[19] Stuber A., Nakatsuka N. (2024). Aptamer Renaissance for Neurochemical Biosensing. ACS Nano.

[20] Mohammadinejad A., Gaman L.E., Aleyaghoob G., Gaceu L., Mohajeri S.A., Moga M.A., Badea M. (2024). Aptamer-Based Targeting of Cancer: A Powerful Tool for Diagnostic and Therapeutic Aims. Biosensors.

[21] Muharemagic D., Labib M., Ghobadloo S.M., Zamay A.S., Bell J.C., Berezovski M.V. (2012). Anti-Fab Aptamers for Shielding Virus from Neutralizing Antibodies. J. Am. Chem. Soc..

[22] Chen L., Zhang B., Wu Z., Liu G., Li W., Tang Y. (2023). In Silico discovery of aptamers with an enhanced library design strategy. Comput. Struct. Biotechnol. J..

[23] Song M., Li G., Zhang Q., Liu J., Huang Q. (2020). De novo post-SELEX optimization of a G-quadruplex DNA aptamer binding to marine toxin gonyautoxin 1/4. Comput. Struct. Biotechnol. J..

[24] Ashrafuzzaman M., AlMansour H.A.M., AlOtaibi M.A.S., Khan Z., Shaik G.M. (2021). Lipid Specific Membrane Interaction of Aptamers and Cytotoxicity. Membranes.

[25] Jerabek-Willemsen M., Wienken C.J., Braun D., Baaske P., Duhr S. (2011). Molecular Interaction Studies Using Microscale Thermophoresis. Assay Drug Dev. Technol..

[26] Zuker M. (2003). Mfold web server for nucleic acid folding and hybridization prediction. Nucleic Acids Res..

[27] Boniecki M.J., Lach G., Dawson W.K., Tomala K., Lukasz P., Soltysinski T., Rother K.M., Bujnicki J.M. (2016). SimRNA: A coarse-grained method for RNA folding simulations and 3D structure prediction. Nucleic Acids Res..

[28] Humphrey W., Dalke A., Schulten K. (1996). VMD: Visual molecular dynamics. J. Mol. Graph..

[29] Jeddi I., Saiz L. (2017). Three-dimensional modeling of single stranded DNA hairpins for aptamer-based biosensors. Sci. Rep..

[30] Abraham M.J., Murtola T., Schulz R., Páll S., Smith J.C., Hess B., Lindahl E. (2015). GROMACS: High performance molecular simulations through multi-level parallelism from laptops to supercomputers. SoftwareX.

[31] Maier J.A., Martinez C., Kasavajhala K., Wickstrom L., Hauser K.E., Simmerling C. (2015). ff14SB: Improving the Accuracy of Protein Side Chain and Backbone Parameters from ff99SB. J. Chem. Theory Comput..

[32] Jorgensen W.L., Chandrasekhar J., Madura J.D., Impey R.W., Klein M.L. (1983). Comparison of simple potential functions for simulating liquid water. J. Chem. Phys..

[33] Heyer L.J., Kruglyak S., Yooseph S. (1999). Exploring Expression Data: Identification and Analysis of Coexpressed Genes. Genome Res..

[34] Fedorov D.G., Kitaura K. (2004). The importance of three-body terms in the fragment molecular orbital method. J. Chem. Phys..

[35] Gaus M., Goez A., Elstner M. (2013). Parametrization and Benchmark of DFTB3 for Organic Molecules. J. Chem. Theory Comput..

[36] Nishimoto Y., Fedorov D.G. (2016). The fragment molecular orbital method combined with density-functional tight-binding and the polarizable continuum model. Phys. Chem. Chem. Phys..

[37] Fedorov D.G. (2017). The fragment molecular orbital method: Theoretical development, implementation in GAMESS, and applications. WIREs Comput. Mol. Sci..

[38] Schmidt M.W., Baldridge K.K., Boatz J.A., Elbert S.T., Gordon M.S., Jensen J.H., Koseki S., Matsunaga N., Nguyen K.A., Su S. (1993). General atomic and molecular electronic structure system. J. Comput. Chem..

[39] Tolosa L., Donato M.T., Gómez-Lechón M.J. (2015). General Cytotoxicity Assessment by Means of the MTT Assay. Methods Mol. Biol..

[40] Yuan S., Zhang N., Singh K., Shuai H., Chu H., Zhou J., Chow B.K.C., Zheng B.-J. (2015). Cross-protection of influenza A virus infection by a DNA aptamer targeting the PA endonuclease domain. Antimicrob. Agents Chemother..

[41] Choi S.K., Lee C., Lee K.S., Choe S.-Y., Mo I.P., Seong R.H., Hong S., Jeon S.H. (2011). DNA aptamers against the receptor binding region of hemagglutinin prevent avian influenza viral infection. Mol. Cells.

[42] El Deeb S., Al-Harrasi A., Khan A., Al-Broumi M., Al-Thani G., Alomairi M., Elumalai P., Sayed R.A., Ibrahim A.E. (2022). Microscale thermophoresis as a powerful growing analytical technique for the investigation of biomolecular interaction and the determination of binding parameters. Methods Appl. Fluoresc..

